# Self-care advice for patients after surgery for oesophageal cancer — a mixed-methods systematic review

**DOI:** 10.1007/s11764-024-01551-0

**Published:** 2024-02-15

**Authors:** Anna Schandl, Kenneth Färnqvist, Kalle Mälberg, Sandra Nielsen, Pernilla Lagergren

**Affiliations:** 1 Surgical Care Science, Department of Molecular Medicine and Surgery, Karolinska Institutet, Retzius Väg 13A, 4th Floor, 17177 Stockholm, Sweden; 2 Department of Anaesthesia and Intensive Care, Södersjukhuset, 11883 Stockholm, Sweden; 3 Department of Clinical Science and Education, Karolinska Institutet Södersjukhuset, 11883 Stockholm, Sweden; 4 Department of Surgery and Cancer, Imperial College London, London, UK

**Keywords:** Cancer survivorship, Symptoms, Functions, Self-management

## Abstract

**Purpose:**

The aim of the literature review was to identify and synthesise research on self-care advice for oesophageal cancer survivors.

**Methods:**

A mixed-methods systematic review and synthesis of existing literature on the topic. Five databases were searched for studies providing information on self-care advice for survivorship after oesophageal cancer surgery, in English, with no time filter. The Critical Appraisal Skills Program was used to assess the risk of bias. Data were presented by textual descriptions and grouping of data.

**Results:**

Among the 13 studies included in the review, five pieces of self-care advice were identified; reconstructing eating habits, bed-head elevation, health-promoting advice, monitoring symptoms and body functions, and involving family and friends. The self-care advice was experienced to be hard work, but worth the effort. They also provided reassurance and an increased understanding of bodily changes and social consequences of the disease and treatment.

**Conclusions:**

There are is little evidence-based self-care advice for oesophageal cancer survivors. However, the existing self-care advice was appreciated and contributed to an increased understanding of the situation. Comprehensible and easy-to-follow recommendations should be provided to all oesophageal cancer survivors.

**Implications for Cancer Survivors:**

Evidence-based self-care advice helpful for the individual oesophageal cancer survivor may be imperative to cope with the consequences of oesophagectomy after hospital discharge.

**Supplementary Information:**

The online version contains supplementary material available at 10.1007/s11764-024-01551-0.

## Background

Self-management is an essential component of care for cancer survivors [1]. After treatment, most patients are provided with recommendations on how to manage symptoms, cope with potential side effects, and where to turn if problems arise [2]. However, detection of complications may rely on the patients’ ability to make a distinction between expected and concerning symptoms and to contact appropriate clinical healthcare services outside of routine clinical appointments [3]. Patients who have undergone curatively intended surgical treatment for oesophageal cancer often have a long recovery time ahead of them. The surgical procedure is extensive and involves removing large parts of the oesophagus and thereafter forming parts of the stomach into a tube as a replacement for the oesophagus [4]. Literature demonstrates that oesophageal cancer resection has a major negative impact on health-related quality of life, most pronounced during the first postoperative year, but also from a more long-term perspective [5–8]. Detriments in physical, role, and social function, as well as difficulties eating, reflux, and fatigue, are common in the aftermath [9–11]. The permanent anatomical changes of the oesophagus often require life-long lifestyle changes [12]. Self-care advice may help to manage chronic conditions, relieve illness burden, and help patients regain a sense of control of the situation [13–15]. Self-care can be defined as “The ability to care for oneself through awareness, self-control, and self-reliance to achieve, maintain or promote optimal health and well-being” [16]. However, the understanding and use of the term “self-care” varies between studies but generally reflects the role that a person takes in monitoring and managing their own health. In this study, self-care was regarded as a more comprehensive concept involving self-management and self-monitoring occurring in the context of a health condition with or without input from healthcare professionals [17]. A systematic review and meta-analysis on the topic indicated that self-management interventions had positive effects on cancer survivors’ quality of life [18]. However, the majority of the 12 studies included in the meta-analysis were small single-centre studies and the study population consisted mostly of breast cancer survivors. Specific self-care advice may vary between cancer diagnoses and to our knowledge, no other research to date has examined self-care advice for oesophageal cancer survivors. Therefore, the aim of the literature study was to identify and synthesise research on self-care advice for oesophageal cancer survivors. The study may provide valuable insights that may result in changes in health service provision.

## Methods

### Study design

A mixed-methods systematic literature review was conducted following a systematic methodology for various study types [19, 20]. The research question could be addressed by quantitative as well as qualitative research designs and therefore a convergent integrated approach for synthesis and integration was followed [21].

### Search strategy

The literature search strategy was developed and agreed upon by the researchers and was consulted with librarians at Karolinska Institutet. An electronic search was conducted with assistance from Karolinska Institute University Library for articles reporting self-care advice for oesophageal cancer survivors and experiences for this advice in the five databases: PubMed, CINAHL, Cochrane, Web of Science, and Scopus. The original search was conducted on 13 December 2021 and was updated on 1 September 2023. The search strategy is shown in Supplementary Table 1. Reference lists of included articles were also examined to identify potential studies that were not found through the database searches, but eligible for inclusion. To ensure that the results were as comprehensive as possible, references from the studies included were traced. For an article to be eligible for this study, it had to fulfil the following inclusion criteria:

- Quantitative, qualitative, or mixed-methods studies
- Patients had undergone oesophagectomy for oesophageal cancer
- Include self-care advice for oesophageal cancer survivors
- The self-care advice could be provided in a hospital or practiced at home

Studies were excluded if they were systematic reviews, conference abstracts, case reports, or protocols. Language was limited to English, and studies had to be published as full-text articles to be eligible.

### Study selection

**Fig. 1:**
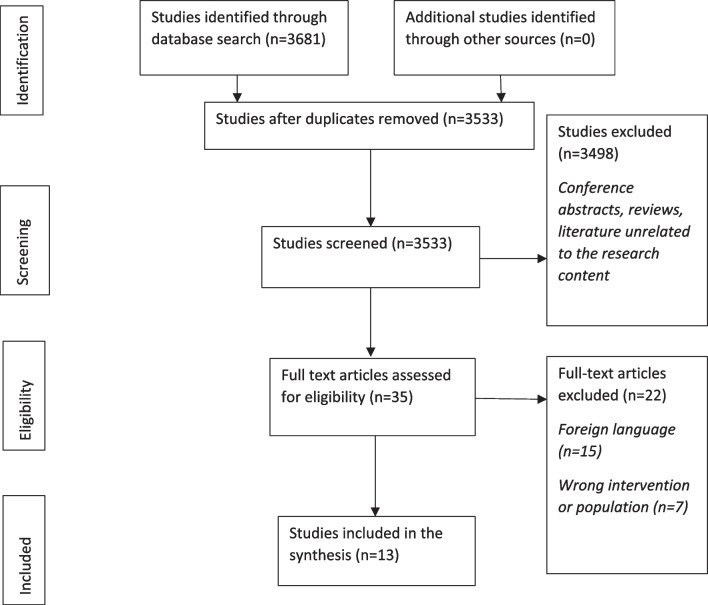
Flow diagram of studies included and excluded at each stage of the review

The selection was based on article titles performed by two researchers (SN, AS). Abstracts and full texts were independently assessed by two researchers (SN, AS). A third researcher was available to resolve potential discrepancies between the two reviewers; however, this was not required. The screening process of the literature is presented in Fig. 1. A total of 3681 studies were preliminary retrieved and 3533 were left after the removal of duplicates. According to the exclusion criteria case reports, conference abstracts, study protocols, reviews, and literature unrelated to the research content, 3498 papers were removed, with 35 remaining. After further reading the full text of the remaining literature, we excluded those of foreign language and with the wrong population or intervention. In total, 22 papers were excluded, leaving 12 studies for further analysis. The updated literature search rendered one more relevant study. In total, 13 studies underwent evaluation of the methodological quality and were included in the study. Data were independently extracted by three researchers (KF, KM, AS) using data extraction forms. Exact words were extracted without interpreting data.

### Methodological quality

Eligible studies were critically appraised by three independent researchers (KM, KF, AS) for methodological quality using The Critical Appraisal Skills Program (CASP) [22]. The evaluation content involves 10–12 items depending on the study design, each item evaluated by “yes”, “no” or “cannot tell”. The studies were graded A-C, depending on whether the study met the appraisal standards or not. Grade A indicated the lowest possible risk of bias. Grade B was used for studies with a moderate possibility of bias, i.e., partially missing quality standards. Grade C was rewarded if several quality standards were not met, and the study was regarded as the highest possibility of bias. Disagreements were discussed among the researchers until a consensus was reached. All studies were included in the study independent of grading results.

### Data extraction

Three researchers independently extracted quantitative and qualitative data from the included studies using an Excel data extraction tool. The usefulness and appropriateness of the extraction form were tested on two papers and were found applicable. The extracted data included details about the population, context, research design and study methods, and information on self-care advice. For qualitative papers, the results section was read several times to fully immerse in the data, and experiences of self-care advice were extracted. Any disagreements were resolved through discussion. To involve patient perspectives, the results were then discussed with patients who had undergone oesophagectomy for cancer in recent years.

### Data synthesis

Because of the heterogeneity of the studies’ aim, population, and methods as well as differences in reported outcomes, a narrative synthesis of the results was undertaken. Quantitative findings were “qualitised”, which involves extracting data from quantitative studies and translating it into textual descriptions to allow integration with qualitative data [21]. Thus, the quantitative results are narratively interpreted [21]. In this study, qualitised data comprised describing self-care advice (as study interventions) or self-rated outcomes (experiences) expressed as word categories. The transformed textual descriptions were then brought together with the qualitative data extracted directly from the qualitative studies. The researchers undertook repeated, detailed examination of the assembled data and identified groups/themes based on similarity in meaning. The themes were continuously reviewed and compared to identify similarities and differences. Each theme could therefore include qualitative or qualitised data or both. The themes were combined to produce a description of the overall findings of the literature review. This method of analysis is in concordance with an inductive thematic analysis approach [23]. The findings were presented as textual descriptions of the grouped data.

## Results

### Included studies

Among the 13 included studies, a total of 367 patients (250 men and 117 women) from Ireland, United Kingdom, the United States, China, Taiwan, Japan, Denmark, and Sweden reported self-care advice (Table 1). Six out of the 13 studies were of quantitative design [24–29], one randomised controlled trial [26], two non-randomised studies [28, 29], two cohort studies [24, 27], and one cross-sectional study [25]. All studies (6/6) received grade C on the appraisal tool indicating a relatively high risk of bias [24–29]. One publication was a mixed-method study [30] of which only the qualitative part was included for analysis. The study was graded B, representing a moderate risk of bias. Six of 13 studies were of qualitative design [31–36] and included descriptive qualitative research (*n* = 3) [31, 33, 36], phenomenology (*n* = 1) [32], phenomenology – hermeneutics (*n* = 1) [34], and grounded theory (*n* = 1) [35]. Among the qualitative studies, one study rated grade A [32], four grade B [31, 33–35], and one grade C [36]. The detailed critical appraisal scoring can be found in Table 2.

**Table 1:** Characteristics of the individual studies included in the review

Author/year	Country	Participants (male/female)	Research design	Data collection	Aims	Type of advice	Outcomes
Bennett (2020) [31]	Ireland	*n* = 18 (14/4)	Qualitative thematic analysis	Focus group interviews with patients who had undergone oesophagectomy within 5 years	To explore the experiences of oesophageal cancer diagnosis, treatment, and recovery focusing on health education needs	Personal advice	Patients experienced: -Lack of understanding of what to expect throughout treatment and recovery - The demanding period of adjustment required because of changes in physical, mental, and social functioning - Support provided by family, and friends was variable and uninformed
Cheng (2020) [24]	China	*n* = 20 (18/2)	Single-armed, non-randomised pilot study	Assessment of depression, anxiety, stress, nutrition, and physical fitness before surgery, 1 and 3 months after oesophagectomy	To evaluate the feasibility, safety, and efficacy of an intervention model using a mobile health system	The programme included guidelines on postoperative complications, nutrition, physical exercise promotion, and psychological support courses	The programme was shown to be feasible, usable, and safe
Eng (2018) [25]	USA	*n* = 31 (20/11)	Cross-sectional design	Quality of life symptoms, and diet adjustments were assessed with questionnaires 6–12 months after oesophagectomy or gastrectomy	To evaluate patients’ quality of life, symptoms, and self-management strategies	Self-reported advice	Patients reported the following advice: To chew food completely, eat smaller meals, control the meal portions, sit upright after eating, keep upper body raised when sleeping, and plan social activities around eating
Huang (2021) [26]	Taiwan	*n* = 14 (13/1)	RCT 2-week crossover design	Reflux symptoms were assessed with a questionnaire and endoscopic observations in patients who had undergone oesophagectomy with a minimum of 2 months	To examine the effect of head-of-bed elevation by using a wedge-shaped pillow on reflux	Bed-head elevation	Combined drug and use of a wedge-shaped pillow provide a more significant easing of the reflux symptoms over 3 months than drug-only treatment
King (2023) [30]	Ireland	*n* = 14 (9/5)	Mixed-method design	Fatigue, anxiety, depression, and quality of life were assessed with questionnaires + focus groups- and individual interviews with patients who had completed treatment for oesophageal cancer	To explore the feasibility of OptiMal for oesophageal cancer survivors	Six group-based sessions of self-management support programme for handling persistent symptoms	Fatigue, difficulty performing usual activities, anxiety, and depression were reduced at 3 months follow-up. Patients reported applying self-management strategies to improve their well-being
Komatsu (2018) [27]	Japan	*n* = 29 (27/2)	Single-armed cohort study	Physical activity, BMI, psychological distress, and quality of life were measured with questionnaires pre-surgery, 2–4 weeks, and 3 and 6 months after oesophagectomy	To describe changes in physical activity, BMI, psychological distress, and quality of life in patients receiving nurse counselling for physical activity	Nurse counselling included information about postoperative complications, theory, and practice of physical activity	Postoperative physical level returned to nearly preoperative level and the number of inactive patients decreased to half at 6 months
Missel (2018) [32]	Denmark	*n* = 10 (8/2)	Phenomenological study	Individual interviews 4 weeks after oesophageal resection	To provide insight into patients’ lived experiences of participating in an education and counselling nutritional intervention	Four sessions of nurse counselling and education to change eating habits while adapting to their new oesophagus	Patients experienced -Embodied disorientation -Living with increased attention to bodily functions -Re-embodying eating
Nielsen (2021) [33]	Sweden	*n* = 60 (43/17)	Qualitative inductive design	Questionnaire-based with open-ended questions 1 year after oesophagectomy	To examine the advice that patients would like to share with future patients, based on personal experiences	Personal advice	Patients reported the following advice: Health-promoting advice -Acknowledging the new situation -Embracing support from others
Richards (2020)[28]	United Kingdom	*n* = 40 (27/13)	Pilot study	Electronic symptom report at hospital discharge, twice the first week, weekly post-discharge in patients who had undergone upper gastrointestinal surgery for cancer	To evaluate the feasibility of a real-time electronic symptom monitoring system after hospital discharge	Tailored self-management advice, prompts to contact a clinician or automated clinician reports depending on symptom severity	The ePRO system was found reassuring, providing timely and relevant information and advice
Sjeltoft (2020) [34]	Denmark	*n* = 13 (7/6)	Phenomenological-hermeneutical study	Individual interviews with patients who had undergone oesophagectomy within 1 year	To explore the lived experience of patients in terms of eating and consequences in everyday life in the first year after oesophageal cancer surgery	Experiences	Patients experienced: Adjusting to the different anatomy-food and eating as a dominant and difficult activity Changed body — food and eating as an underlying shadow Feeling different — social consequences of changed eating A nutritional jungle — guidance and support in an uncertain time
Ueda (2020) [35]	Japan	*n* = 12 (9/3)	Grounded theory approach	Individual interviews with patients from 3 months onwards after oesophageal cancer surgery	To identify the eating behaviours of patients from 3 months onwards after oesophageal cancer surgery	Advice related to eating behaviours	The patients go through three stages with respect to eating behaviours during the first year of surgery: -To learn how to swallow -How to cope with the symptoms occurring during eating -To build self-management skills
Wainwright (2007) [36]	United Kingdom	*n* = 11 (8/3)	Qualitative descriptive design	Semi-structured individual interviews with patients who had undergone oesophagectomy for cancer over 10 months and replied “quite a bit” or “very much” in a quality of life questionnaire	To gain a more in-depth understanding of patients’ experience of appetite loss and how this affected their quality of life	Monitoring of weight and increase food intake	Patients described: -The meaning of weight loss and physical change -Remapping the body -Eating as a social activity
Zhang (2021) [29]	China	*n* = 124 (74/50)	Comparison study (non-randomised)	The self-care ability score, social relationship quality score, mood state score, hope-level score, and nursing job satisfaction were evaluated in individuals with oesophageal cancer after surgery	To explore the effect of rehabilitation-oriented nursing intervention on self-care ability and social relationship quality	Rehabilitation-oriented nursing intervention	The intervention was associated with lower scores of depression and anxiety and higher scores on self-care ability, hope level, and social relations

**Table 2:** Methodological quality appraisal of each included study

	1	2	3	4	5	6	7	8	9	10	11	12	Quality
Bennett (2020) [31]	Y	Y	Y	Y	Y	Y	C	Y	Y	Y			B
Cheng (2020) [24]	Y	Y	Y	Y	N	Y	N/A	N/A	N	N	N	N	C
Eng (2018) [25]	Y	Y	N	Y	N	Y	N/A	N/A	N	N	N	N	C
Huang (2021) [26]	Y	N	Y	N/C/C	Y	Y	Y	Y	Y	N	Y		C
King (2023) [30]	Y	Y	Y	Y	Y	N	Y	C	C	N/A			B
Komatsu (2018) [27]	Y	Y	Y	Y	C/C	Y/Y	N/A	N/A	N	N	Y	C	C
Missel (2018) [32]	Y	Y	Y	Y	Y	Y	Y	Y	Y	Y			A
Nielsen (2021) [33]	Y	Y	Y	Y	Y	C	C	Y	Y	N/A			B
Richards (2020) [28]	Y	Y	Y	Y	N/N	Y/Y	N/A	N/A	N	N	N	Y	C
Sjeltoft (2020) [34]	Y	Y	Y	Y	Y	N	Y	Y	Y	N/A			B
Ueada (2020) [35]	Y	Y	Y	N	Y	N	Y	Y	Y	N/A			B
Wainwright (2007) [36]	Y	Y	Y	C	Y	N	N	Y	Y	N/A			C
Zhang (2021) [29]	Y	Y	Y	Y	N	C	N/A	N/A	N	N	Y	C	C

*Y* yes; *N* no; *C* cannot tell; *N/A* not applicable

### Available self-care advice

All included studies reported self-care advice either provided by healthcare such as symptom-reducing devices (wedge-shaped pillow) [26], educational programmes [29, 30], counselling interventions [27], or advice based on patients own experiences [25, 31–36] or obtained through digital platforms [24, 28]. The identified self-care advice was categorised according to content and included the following: reconstructing eating habits, bed-head elevation, health-promoting advice, monitoring symptoms and body functions, and involving family and friends (Table 3).

**Table 3:** Overview of the result presentation

Available self-care advice	Experiences of self-care advice
Reconstructing eating habits	Hard work, but worth the effort
Bed-head elevation	Reassurance
Health-promoting advice	Understanding the bodily changes
Monitoring symptoms and body functions	Understanding the social consequences
Involving family and friends	

#### Reconstructing eating habits

A common self-care advice for oesophageal cancer survivors was suggestions on how to adapt eating behaviours to the new circumstances [29, 30, 32]. Some advice included healthcare interventions, such as education of patients regarding the importance of adequate nutrition to maintain a healthy body weight, but also providing information on physical, mental, and social problems related to eating difficulties [24, 32]. Creating individual nutrition plans [24] and goal-setting activities [30] followed by repeated goal reviews were suggested as means to facilitate the implementation and sustainability of self-management skills [30, 32].

Other advice on how to change eating habits was based on personal experiences. This advice was more hands-on and included recommendations on how to manage the situation in daily life [25], such as to eat smaller portions of food at frequent intervals (to compensate for the small amount) [25, 33–36], to chew well, make small bites and eat at own pace [25, 35], to have vomit bags close by [33], to choose certain types of food which contain more protein and nutrients [34], instructions on how to cook to keep the nutritive values [34, 35], to identify own postures and behaviours that facilitate digestion [25, 35], to be careful of oral aspiration and reflux [35], and to plan social activities around eating [25]. Another pragmatic piece of advice was to order a couple of starters or a child’s portion rather than an adult portion of food while eating at restaurants [36]. Willpower and self-control were stated as necessary for maintaining the stability of eating behaviours [35]. The importance of learning how to make a cognitive link between the quantity, type, and timing of food consumption was emphasised in order to maintain adequate weight and health status [36].

##### Bed-head elevation

For those who suffered from burdensome nocturnal reflux symptoms after oesophagectomy, bed-head elevation was advised. One study evaluated a wedge-shaped pillow which supported the patient’s body from the waist to the head to reduce gastrointestinal reflux [26]. The hypothesis was based on that by increasing the head-of-bed elevation, the gravity assistance effect would be restored, and the frequency and severity of gastro-oesophageal reflux reduced.

##### Health-promoting advice

To improve overall quality of life while recovering after oesophagectomy, several general health-promoting advice were suggested. Recommended activities to increase physical strength and stamina were, e.g., inspiratory muscle training, walking exercise, and qigong [24]. Strategies to manage fatigue were also mentioned, but not specified [30]. Two publications advised on how to overcome barriers to physical activity through counselling sessions motivating patients to reduce inactivity [27, 30]. Another health-promoting advice consisted of health education and information about disease and pain management [29]. One study recommended strategies for optimising mental health but the content was not described in detail [30], while another study recommended maintaining a pragmatic and positive attitude [31]. Other advice to overcome post-surgical difficulties were to have patience, be persistent about getting better, and sustain difficulties through determination and a sense of grit [33] or when needed, join psychological support programmes to relieve potential negative emotions [24, 29]. Some advice also highlighted the value of humour to protect well-being [31] and to not let the illness or consequences of surgery get in the way of doing things that were practiced before the cancer. Going back to work and likeable leisure activities were described as an important part of their recovery process [33].

##### Monitoring symptoms and body functions

While adapting to the new anatomy, monitoring symptoms and changed body functions were perceived useful in recovery from disease and the return to normal life. The monitoring could be simple such as monitoring body weight on a scale and following the measurements over time. The advice was to couple the results with strategies for increasing food intake to improve body weight [36]. There were also more advanced methods to monitor symptoms, e.g., electronic patient-reported outcome systems where clinically derived algorithms provided patients with tailored self-management advice and prompts to either contact a clinician or automated clinician alerts depending on symptom severity [28].

##### Involving family and friends

Encouragement, comfort, and support from family members and friends were found to contribute to creating a safe and helpful environment for the oesophageal cancer survivors [29, 32]. Embracing support from others, both from family and friends, but also from healthcare was advice expressed in two studies [32, 33]. To be open and talk about the situation was perceived to increase other peoples’ understanding of the mood or feelings that were experienced during the illness and treatment [33]. One study offered educational sessions on effective communication for use when talking to healthcare professionals, employers, and family members [30]. Not being alone with thoughts and concerns and receiving social support had for many been helpful in handling difficult emotions [33], as stated by one family member “You shouldn’t go through this alone” [32]. The spouses were considered to play an important role in determining postoperative strategies since they could help with complying to the dietary advice and act as food purchasers and/or preparers of meals [36]. They could also assist with practicalities like remembering advice and making decisions [32].

### Experiences of self-care advice

Seven out of 13 studies included patients’ experiences of the suggested self-care advice [30–36]. In general, self-care advice was perceived to be of help in coping with the disease and life after surgery. The advice was perceived to be hard work, but worth the effort. They also provided reassurance and an increased understanding of the bodily changes and the social consequences of the disease and treatment (Table 2).

#### Hard work, but worth the effort

Some of the advice, such as being physically active to prevent loss of muscle mass and fatigue was perceived as exhausting and sometimes even painful. However, it was regarded to be worth the trouble since they gained more energy which allowed them to get back to normal leisure and work activities after a time of illness [30, 33]. It was stated that following the self-care recommendations required ongoing planning. [34]. Meals had to be scheduled and planned with regard to content, size, and timing according to the consequences it had on e.g., nausea and/or bowel movements [34]. Patients stated that they had to actively think about eating to avoid feeling unwell if they ate too little since they did not feel hunger the same way as before [34].

#### Reassurance

The self-care advice provided information on how to manage symptoms while being at home. The positive benefits included increased confidence, motivation, resilience, and control over one’s own health [30]. To receive advice and support from healthcare professionals was described as a relief from thinking or worrying about something out of your control [24, 33]. They found that an electronic patient-reported outcome report system was useful in confirming that their symptoms were typical for their stage of recovery but could also be beneficial in reminding them to follow advice they might have forgotten or to reinforce the guidance they received from healthcare. It also helped in overcoming feelings of isolation and uncertainty following hospital discharge. They spoke about the reassurance they gained from being able to objectively see that their symptoms were improving [28]. Related feelings of reassurance were described by patients who received social support through an online support community via a chat feature [24]. Others described reassurance in discussing symptoms with other patients [30]. Support groups consisting of oesophageal cancer survivors were proposed since learning from those in a similar situation was believed to be beneficial [30, 31, 34].

#### Understanding the bodily changes

Patients described that gaining more knowledge and a deeper understanding of the disease and treatments through counselling or education meant that they understood themselves and their bodily changes better [32]. The knowledge gained enabled an acceptance of their health needs and the long-term effects of cancer and treatment [30]. This was perceived to build a platform that permitted them to start building self-management skills [35]. Strategies for energy or fatigue management were reported to facilitate applying learning to everyday activities [30]. Hands-on advice on what, when, and how to eat when experiencing no appetite [34], or what to be aware of in relation to food and eating [35], were stated to be readily useful. Dietary consultations were found to be supportive during the period of insecurity just after surgery and discharge from hospital [34]. Conversations about diet and nutrition challenges and concerns with healthcare professionals helped patients to verbally describe their experiences of altered bodies [32]. Many survivors’ goals were to gain control over their bodies so they could trust that it would not let them down in public [36].

#### Understanding the social consequences

Eating after oesophageal resection was experienced as different from previous eating habits and quite unlike other people’s eating practices [32]. The new regimens of different foods and portion sizes not only affected the patient but influenced the family routines of eating [36]. Since many patients avoided eating out or eating together with others, the social relations had to be renegotiated which could disturb family and social life [36]. Some patients had a diet plan which was described to create a sense of security and control, but also gave rise to a sense of community and understanding about the impact the illness and treatment had on the patient’s social circle, between patients and their families [32].

## Discussion

This mixed-methods systematic review, including 13 studies, indicates that most self-care advice for oesophageal cancer survivors is of a general character on how to cope with a cancer diagnosis or to maintain a healthy lifestyle. In contrast, more specific advice related to how to manage life with the new oesophagus is less common. Most advice was not evidence-based but shared by other survivors. These findings indicate a need to systematically examine and evaluate the effectiveness of self-care advice provided to patients post-surgery.

Strengths of the study include a priori-defined study protocol, an extensive and systematic literature search performed by an experienced librarian covering a variety of studies of different study designs, and an assessment of different sources of bias. There are also some limitations. The included studies, especially those of quantitative design, had a high risk of bias according to the quality assessment tool. There was high heterogeneity across studies and some advice was included in multimodal programmes which made it difficult to specifically point out if there was any specific advice that was more beneficial. Further, since the literature search was limited to publications in English, studies on self-care advice in other languages are missing. Finally, publication bias for studies evaluating self-care advice after oesophageal cancer resection cannot be ruled out.

Self-care is increasingly regarded as a necessary component of chronic disease and secondary prevention [37]. To be efficient, self-care interventions should be tailored to the patient’s needs and include strategies to improve the patient’s disease or treatment knowledge, independent symptom monitoring, encouragement of self-treatment, psychological coping, and stress management strategies, but also the possibility of receiving feedback from healthcare [38]. Even though some of the self-care advice in this study were in line with health lifestyle recommendations found in the ESPEN practical guidelines for cancer survivors [39] and home enteral nutrition [40], self-care advice is often not systematically developed, provided, or evaluated [41]. To the best of our knowledge, the current study is the first to identify and summarise available self-care advice for use in the first years after oesophagectomy for cancer, whereas existing studies have focused on identifying information and support needs during either the pre-operative phase [42] or at the first follow-up consultation [43]. Many oesophageal cancer survivors struggle daily with the impact of the changed anatomy and subsequent changes in eating years after surgery [12]. Still, the present study identified mostly self-care advice of general character and not adapted to their specific situation. This finding was confirmed in a scoping review including various groups of cancer survivors, where follow-up on dietary changes and support were reported to be limited during survivorship and provided advice were likely to be of generic lifestyle character [44]. This finding may be explained by that clinicians have limited knowledge of evidence-based dietary guidelines or inadequate training to provide health behaviour counselling [45, 46], which is unfortunate since dietary advice is a key component of cancer care beyond the initial recovery from treatment [39].

The findings of this study were discussed with a group of patient representatives of diverse backgrounds who have undergone surgery for oesophageal cancer in recent years [47]. Based on their experiences, the self-care advice varied greatly across hospitals. Some patients seemed to have received a large amount of self-care advice through information leaflets, or in contact with physicians, nurses, or dieticians at follow-up visits while others received none. However, sometimes the terminology was perceived to be difficult to understand for patients and relatives. Thus, the patient representatives expressed the importance that the self-care advice was conveyed in layman’s language. Further, they stated that symptoms change with time, and therefore most probably the need for self-care advice as well. There may be individual preferences, some advice is important for some patients, while others would like to test for themselves. From their perspective, the results of this study were encouraging, but they noticed that there was little self-care advice regarding mental recovery and sleep, which are two important areas for recovery. They also warranted more interactive discussions with healthcare professionals regarding the calibration of medicines, e.g., for reflux or sleep, to reach an optimal drug effect. Lastly, they reflected that much of the self-care advice was based on clinical or personal experiences and not on research or evidence and suggested these as key areas of future research. By applying patients’ views on study results, the clinical relevance of the research improves. Still, it must take into consideration that individual opinions may not be taken as representative of the whole patient population.

To improve readiness and clarify what is expected of patients after hospital discharge, it is crucial to have access to evidence-based recommendations that may help alleviate burdensome symptoms and manage everyday life. However, up to 93% of patients with cancer report dissatisfaction with the way information is provided [48]. Education programmes, including follow-up meetings after surgery for patients and their families, could be one option to ensure receiving valuable information, contacts, and tools to improve survivorship. Furthermore, monitoring of symptoms, problems, and side effects after oesophageal cancer treatment with individualised guidance and support for self-care actions may be a future tool of great value for these patients. This initial review may lead to studies examining the effectiveness of self-care advice provided to patients post-surgery, as well as their influence on patient autonomy and quality of life and potentially guide intervention studies on self-management where lacking. Future studies on this topic are warranted because more high-quality cohort studies and randomised controlled trials would improve the status of the current evidence.

## Conclusion

The study indicates that most self-care advice for oesophageal cancer survivors was of a general character on how to cope with cancer and its treatment effects. Most advice was shared by other survivors but appreciated and perceived to contribute to an increased understanding of the situation. However, given the limited number and quality of studies published, the effectiveness of the self-care advice is not clear, and evidence from large prospective studies is needed.

## Supplementary information

Below is the link to the electronic supplementary material.

Supplementary file1 (DOCX 12 KB)

## Data Availability

The data that support the findings of this study are available upon request by the corresponding author (AS).
