# Supplementary material for: Self-care advice for patients after surgery for oesophageal cancer — a mixed-methods systematic review
Source: J Cancer Surviv. 2024 Feb 15;19(4):1272–84. doi: 10.1007/s11764-024-01551-0 (PMC12283782; doi:10.1007/s11764-024-01551-0)
Supplement: Supplementary file 1 — Supplementary file1 (DOCX 12 KB) [file 11764_2024_1551_MOESM1_ESM.docx]

**SUPPLEMENTARY TABLE 1. THE SEARCH STRATEGY**

Search terms: “self-management”, “self-managing”, “self-managed interventions” OR “self-care”, “symptom management”, “self-care advice”, “self-care recommendations” AND “oesophageal cancer” OR “oesophageal cancer surgery” OR “post oesophageal cancer/cancer surgery” (interview* OR "patient-recommendation" OR "patient guidelines") AND (("oesophageal OR "esophageal" OR "oesophagus" OR "esophagus") AND cancer AND surgery)
